# Effect of Volume Fraction of Reinforcement on Microstructure and Mechanical Properties of In Situ (Ti, Nb)B/Ti_2_AlNb Composites with Tailored Three-Dimensional Network Architecture

**DOI:** 10.3390/ma15249070

**Published:** 2022-12-19

**Authors:** Ningbo Zhang, Boyu Ju, Taiqing Deng, Sen Fu, Cungao Duan, Yiwei Song, Yijun Jiang, Qin Shen, Caogen Yao, Mingda Liu, Ping Wu, Ziyang Xiu, Wenshu Yang

**Affiliations:** 1 Aerospace Research Institute of Materials & Processing Technology, Beijing 100076, China; 2 School of Materials Science and Engineering, Harbin Institute of Technology, Harbin 150001, China; 3 Shanghai Radio Equipment Research Institute, Shanghai 200050, China; 4 Shanghai Research Institute of Radio Equipment, Shanghai 200090, China; 5 CASIC Space Engineering Development Co., Ltd., Xinzhou 431400, China; 6 Key Laboratory of Advanced Science and Technology on High Power Microwave, Xi’an 710024, China; 7 Northwest Institute of Nuclear Technology, Xi’an 710024, China

**Keywords:** metal-matrix composites, Ti_2_AlNb, microstructure, mechanical properties, TiB short fiber

## Abstract

The mechanical properties of (Ti, Nb)B/Ti_2_AlNb composites were expected to improve further by utilizing spark plasma sintering (SPS) and inducing the novel three-dimensional network architecture. In this study, (Ti, Nb)B/Ti_2_AlNb composites with the novel architecture were successfully fabricated by ball milling the LaB_6_ and Ti_2_AlNb mixed powders and subsequent SPS consolidation. The influence of the (Ti, Nb)B content on the microstructure and mechanical properties of the composites was revealed by using the scanning electron microscope (SEM), transmission electron microscopy (TEM) and electronic universal testing machine. The microstructural characterization demonstrated that the boride crystallized into a B27 structure and the α_2_-precipitated amount increased with the (Ti, Nb)B increasing. When the (Ti, Nb)B content reached 4.9 vol%, both the α_2_ and reinforcement exhibited a continuous distribution along the prior particle boundaries (PPBs). The tensile test displayed that the tensile strength of the composites presented an increasing trend with the increasing (Ti, Nb)B content followed by a decreasing trend. The composite with a 3.2 vol% reinforcement had the optimal mechanical properties; the yield strengths of the composite at 25 and 650 °C were 998.3 and 774.9 MPa, showing an 11.8% and 9.2% improvement when compared with the Ti_2_AlNb-based alloy. Overall, (Ti, Nb)B possessed an excellent strengthening effect and inhibited the strength weakening of the PPBs area at high temperatures; the reinforcement content mainly affected the mechanical properties of the (Ti, Nb)B/Ti_2_AlNb composites by altering the α_2_-precipitated amount and the morphology of (Ti, Nb)B in the PPBs area. Both the continuous precipitation of the brittle α_2_ phase and the agglomeration of the (Ti, Nb)B reinforcement dramatically deteriorated the mechanical properties.

## 1. Introduction

The development of the aerospace industry has placed higher demands on material performance; typically, aerospace engines require a new structural material with greater specific stiffness, specific strength and service temperature [1,2,3]. Ti_2_AlNb alloys, after three decades of development, have emerged as the most promising lightweight structural material serviced at elevated temperature and are predicted to be an alternative to the high-density Inconel 718 superalloy. Although this superalloy possesses higher specific strength than nickel-based superalloys, better plasticity and processability than other Ti-Al alloys (Ti_3_Al and TiAl), and superior high-temperature mechanical properties than Ti-based alloys, it is still difficult to completely replace the Inconel 718 superalloy with the Ti_2_AlNb alloys due to insufficient strength and creep resistance at elevated temperatures [4,5,6,7,8]. Thus, improving the mechanical properties of the Ti_2_AlNb alloy becomes an urgent problem to satisfy its application in engineering.

The addition of ceramic particles, short fiber and whisker into the soft matrix via a specific preparation procedure is an effective method for improving the mechanical properties of Ti_2_AlNb alloys. By the strengthening effect of these stiff reinforcements (such as Ag [9], graphene [10,11], TiC [12,13,14], TiB [15,16,17], etc.), the mechanical [7,8], wear [11], fatigue [4,18] and creep [8] properties of Ti_2_AlNb alloys can be significantly improved without sacrificing or with slight degrading of other properties. Among these reinforcements, boride is the appropriate reinforcement for the Ti_2_AlNb matrix because of its exceptional physical and chemical compatibility with Ti_2_AlNb alloys [8,19]. Emura [7,8] fabricated a 6.5 wt% TiB/Ti-22Al-27Nb composite by hot isostatic pressing (HIP) and subsequent hot rolling using TiB-containing pre-alloyed powder; the fine boride (with a length of less than 5 μm and a width of less than 1 μm) in the composite exhibited an oriented alignment along the rolling direction. The subsequent tensile test revealed that the composite presented an increased strength and elastic modulus along the rolling direction and the TiB addition retarded the strength degradation of the matrix at high temperatures. Cowen et al. [15,20] synthesized a Ti_2_AlNb composite in which the boride presented a length of 158 μm and a width of 22 μm by HIP using the Ti-22Al-26Nb-5B powder as a raw material; the mechanical tests demonstrated that the boride incorporation deteriorated both the tensile and creep properties of the Ti_2_AlNb matrix alloy at 650 °C. By analyzing the fracture surface, the authors concluded that the excessively coarse reinforcement is one of the reasons for the poor mechanical properties.

As a fast and efficient sintering method, SPS has been widely adopted in the fabrication of metal-matrix composites. The process is based on a modified hot-pressing process in which the pulsed current passes directly through the pressing die and powder instead of an external heater, and fast heating and short process cycles are achieved by means of pulsed currents and the so-called “spark plasma effect” [21]. SPS, when compared to other traditional sintering procedures (such as hot-pressing sintering and hot-isostatic-pressing sintering), shortens the soaking time and lowers the sintering temperature, resulting in a finer microstructure and excellent comprehensive mechanical properties for the composite. Using Ti6Al4V micro-powder, B_4_C and B nanopowders, Huang et al. [22,23] fabricated Ti-based composites reinforced by TiC nano-particles and TiB nano-whiskers, in which the reinforcement presented a novel three-dimensional network architecture (3DNA); the subsequent tensile test exhibited that the composites with the nano-scale reinforcements and the novel architecture possessed a significant enhancement in both plasticity and strength compared to the composites with the micro-scale reinforcement and homogeneous distribution. Moreover, Zhang et al. [24] synthesized a TiB/Ti_2_AlNb composite with 3DNA by ball milling and subsequent SPS and found that (Ti, Nb)B exhibited good strengthening effects and that the 1.6 vol% TiB addition increased the yield strength of the Ti_2_AlNb matrix by 8.0% at room temperature.

The volume fraction of the boride reinforcement is a key parameter affecting the microstructure and properties of the Ti-based and Ti_2_AlNb-based composites. It was found that the incorporation of the boride hinders the migration of the β/B2 grain boundary during consolidation, heat treatment and sintering processes, and as a result, the microstructure of the matrix was refined [4,25]. Emura et al. [4] demonstrated that the prior B2 grain size of the Ti-22Al-27Nb alloy was reduced from 160 to 5 μm by the 6.5 wt% TiB addition. The addition of reinforcements also influenced the precipitation behavior of the Ti_2_AlNb matrix. Tang [19] and Hagiwara et al. [25] found that the introduction of the TiB reinforcement promoted the precipitation of the α_2_ phase. Cowen et al. [20] displayed that the addition of 2.0 vol% TiNbB_2_ increased the precipitated amount of the α_2_ phase in the Ti-22Al-26Nb alloy from the original 6 vol% to 20 vol%. The volume fraction of the boride reinforcement could also alter the mechanical properties of these composites. Generally, the volume fraction of reinforcement in composites possesses a critical value: when the reinforcement content is lower than this critical value, the tensile strength of the composites increases with the increase of reinforcement content, while when the volume fraction of the reinforcing phase is higher than this value, the mechanical properties of the composites deteriorate gradually [26,27,28].

Based on the above analysis, it is clear that the volume fraction of the reinforcement is indeed closely related to the microstructure and mechanical properties of the in situ boride-reinforced Ti_2_AlNb composites with the novel 3DNA. Unfortunately, the effect of the volume fraction of boride on the microstructure and mechanical properties of the composite has not been revealed since the boride contents are fixed in the previous research (typically, 6.5 wt% [7,8], 1.6 vol% [24] and 5.5 wt% [15,20]). Moreover, though having higher plasticity than the TiAl and Ti_3_Al alloys, the Ti_2_AlNb alloys are still intrinsically brittle materials, and thus, the additional amount of boride should be limited to a specified range to avoid the premature failure of the boride-reinforced Ti_2_AlNb composite. In view of this, in this study, the (Ti, Nb)B/Ti_2_AlNb composite with 3DNA was successfully synthesized by ball milling and subsequent SPS, and the effect of the volume fraction of reinforcement on the microstructure and mechanical properties of in situ network-strengthened (Ti, Nb)B/Ti_2_AlNb composites was revealed.

## 2. Materials and Experimental Methods

### 2.1. Fabrication of (Ti, Nb)B/Ti_2_AlNb Composites

**Figure 1:**
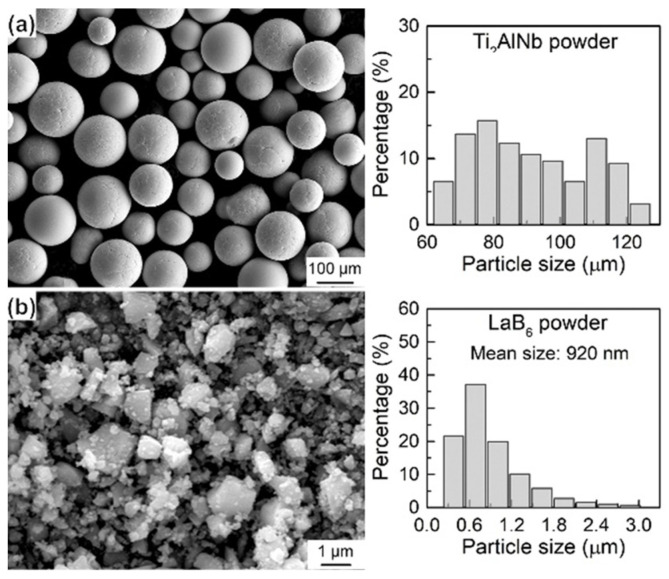
SEM morphology and particle size distribution of raw powders. (**a**) Ti_2_AlNb powder, (**b**) LaB_6_ powder.

The following is a detailed description of the powder metallurgy process used to prepare the (Ti, Nb)B/Ti_2_AlNb composites. First, the spherical Ti_2_AlNb powder, fabricated by the plasma rotating electrode process, and the mechanically crushed LaB_6_ powder were mixed for 8 h at 250 rpm using a QM-3SP2 planetary ball mill with a ball-to-weight ratio of 5:1. The Ti_2_AlNb particles (shown in Figure 1a) are spherical in shape with a bimodal diameter distribution, concentrated between 65.0–124.2 μm. The LaB_6_ particles (as depicted in Figure 1b) are irregular polygons with an average particle size of about 920 nm.

**Figure 2:**
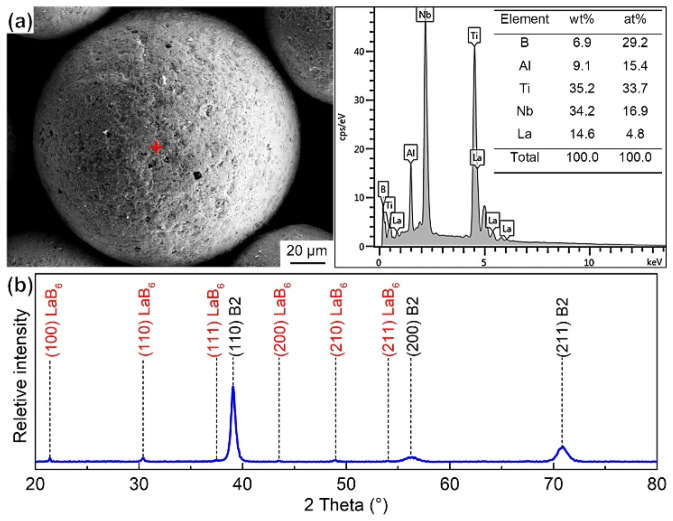
Characterization of Ti_2_AlNb and LaB_6_ mixed powders. (**a**) SEM image and corresponding EDS results, (**b**) XRD patterns.

After ball milling, the surface of the Ti_2_AlNb powder was inset with the irregular LaB_6_, and the diffraction peaks of the LaB_6_ and B2 phases emerged in the XRD pattern of the mixed powder, as shown in Figure 2. The mixed powder was stacked into high-density graphite die with an internal diameter of 50 mm, and then was SPS-consolidated by an FCT HPD-250 furnace under a vacuum environment (the pressure inside the furnace was less than 8Pa throughout the entire sintering process). The sintering temperature, axial pressure and soaking time were fixed at 1250 °C, 45 MPa and 20 min, respectively. The (Ti, Nb)B short fiber reinforcement was synthesized by the chemical reaction between the LaB_6_ and Ti_2_AlNb powders during the sintering process. After sintering, the sintered composites were cooled in the furnace to room temperature.

Due to the limited solid solubility of boron in the Ti_2_AlNb matrix, it is possible to assess the volume fraction of (Ti, Nb)B reinforcement *V*_(Ti, Nb)B_ using the following formula:

(1) $$V_{(\mathrm{Ti},\;\mathrm{Nb})B}=\frac{\frac{{6\omega }_{{\mathrm{LaB}}_{6}}M_{(\mathrm{Ti},\;\mathrm{Nb})B}}{M_{{\mathrm{LaB}}_{6}}\rho_{(\mathrm{Ti},\;\mathrm{Nb})B}}}{\frac{{1-\omega }_{{\mathrm{LaB}}_{6}}}{\rho_{{\mathrm{Ti}}_{2}\mathrm{AlNb}}}+\frac{{6\omega }_{{\mathrm{LaB}}_{6}}M_{(\mathrm{Ti},\;\mathrm{Nb})B}}{M_{{\mathrm{LaB}}_{6}}\rho_{(\mathrm{Ti},\;\mathrm{Nb})B}}}\times 100\%$$

where *ρ* and *M* stand for density and molar mass, respectively. $\omega_{{\mathrm{LaB}}_{6}}$ denotes the mass fraction of the LaB_6_ powder in the mixed powders. According to the crystal structure and composition of the (Ti, Nb)B-phase displayed in Table 1, the molar mass and density of (Ti, Nb)B could be calculated as 81.2 g/mol and 6.08 g/cm^3^, respectively.

By adjusting the LaB_6_ percentage in the mixed powders, four batches of the (Ti, Nb)B/Ti_2_AlNb composites with various reinforcement volume fractions were synthesized. For these four composites, the additive amounts of LaB_6_ were 0, 0.8, 1.6 and 2.4%, and correspondingly, the volume fractions of the (Ti, Nb)B reinforcement were 0, 1.6, 3.2 and 4.9 vol% according to Equation (1).

### 2.2. Material Characterization

A SUPRA55 scanning electron microscope (Berlin, Germany) equipped with an energy dispersive spectrometer (EDS) was used to characterize the microstructure and composition of the characteristic regions of fabricated composites. The samples utilized for the SEM analysis were polished and etched by Kroll’s solution (80 vol% H_2_O + 10 vol% HNO_3_ + 10 vol% HF). For the SEM images captured with a secondary electronic mode, the matrix phases can be distinguished by contrast, and normally, the light, gray and dark areas in the matrix are B2, O and α_2_ phases, respectively [29].

Talos F200X transmission electron microscopy (Hillsboro, America) was employed to analyze the local microstructure and phase structure. The foils used for the TEM analysis were ground to a thickness of 40 μm before being ion milled at 5 eV using the GATAN-695 Ion Beam Thinner. The crystal structure of a specific area is obtained by the index of the diffraction pattern, which was acquired by selected area electron diffraction (SEAD) or by the fast Fourier transform (FFT) of the high-resolution transmission electron microscopy image. The FFT process was conducted by using Digital Micrograph software, and the crystal structure and detailed lattice parameters for the possible phases are listed in Table 1.

**Table 1:** Crystal structure and ideal composition of the main phases in (Ti, Nb)B/Ti_2_AlNb composites [30,31].

Phase	Space Group	Composition (at%)	Lattice Parameters (Å)		
			a	b	c
B2	$Pm\overset{-}{3}m$ (221)	(Ti, Al, Nb) solid solution	3.23	3.23	3.23
α_2_	*P63/mmc* (194)	Ti_3_Al	5.74	5.74	4.64
O	*Cmcm* (63)	Ti_2_AlNb	6.05	9.51	4.67
B27-(Ti, Nb)B	*Pnma* (62)	TiNbB_2_	6.16	3.10	4.65
La_2_O_3_	$P\overset{-}{3}m1$ (164)	La_2_O_3_	3.94	3.94	6.13

The mechanical properties of the (Ti, Nb)B/Ti_2_AlNb composites with various reinforcement volume fractions were evaluated by an INSTRON-8862 electronic universal testing machine (Boston, America) with a fixed axial tensile speed of 0.2 mm/min. To lessen the influence of the initial surface micro-cracks, the dog-bone samples, with a gauge dimension of 12 × 3 × 2 mm^3^, were machined using electric discharge machining and then ground with 1000 grit sandpaper to minimize the effect of the original surface micro-cracks. For the 650 °C tensile tests, the samples were coated with a specific glass anti-oxidation coating to avoid the impact of high-temperature oxidation on the surface and were soaked at 650 °C for 6 min to ensure that the heart of the samples reached the test temperature. The samples were quenched into the water once the fracture occurred, and then the fracture surface was observed by SEM.

## 3. Results and Discussion

### 3.1. Microstructure of (Ti, Nb)B/Ti_2_AlNb Composites

**Figure 3:**
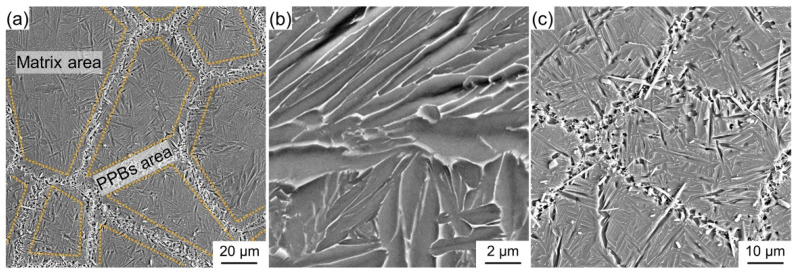
Typical microstructure of (Ti, Nb)B/Ti_2_AlNb composites: (**a**) whole morphology, (**b**) matrix area, (**c**) PPBs area.

**Figure 4:**
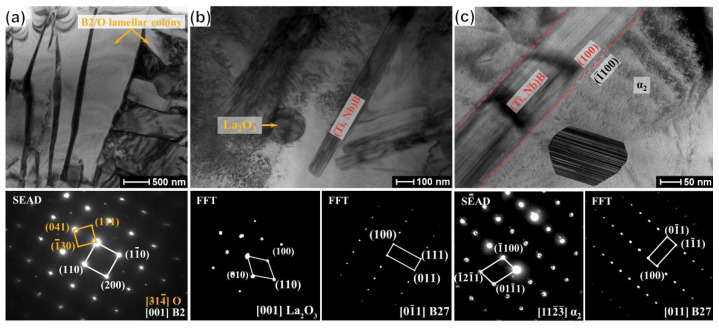
Bright-field image and corresponding SEAD/FFT patterns of 1.6 vol% (Ti, Nb)B/Ti_2_AlNb composite: (**a**) matrix area, (**b**,**c**) PPBs area.

As demonstrated in Figure 3a, the microstructure of the fabricated composites can be divided into two different areas, namely the matrix area and the PPBs area. The (Ti, Nb)B crystallizes into a short fiber morphology and gathers in the PPBs area. The fine LaB_6_ powder is embedded into the surface of the Ti_2_AlNb powder for the mixed powder (see Figure 2), and the (Ti, Nb)B is synthesized by the chemical reaction between LaB_6_ and Ti_2_AlNb, both of which results in the network distribution of (Ti, Nb)B reinforcement. The matrix area is mainly composed of B2 and O phases and a feature of the alternate distribution of lamellar B2 and O phases, as shown in Figure 4a. The α_2_-Ti_3_Al, B2 solid solution and O-Ti_2_AlNb are the main phases for Ti_2_AlNb alloys [5]. The rapid cooling feature of SPS seems to inhibit the precipitation of α_2_, and instead, abundant lamellar O phase (gray area) precipitates from the B2 grain, as demonstrated in Figure 3b.

The bright-field image and diffraction patterns of the PPBs area, depicted in Figure 4b,c, suggest that (Ti, Nb)B crystallizes into a B27 crystal structure and that the by-product La_2_O_3_ and brittle α_2_ phase also emerges in the PPBs area. The α_2_-phase precipitates cling to the (100) surface of the (Ti, Nb)B phase, which implies that the synthesized (Ti, Nb)B promotes the precipitation of the α_2_ phase. For the in situ boride-strengthened Ti-based and TiAl-based composites, the borides usually possess B27 and Bf structures [32,33,34]. It is the same result as Cowen’s that the boride only possesses the B27 crystal structure in the in situ boride-reinforced Ti_2_AlNb-based composites. Zhang et al. [24,35] have demonstrated that the boride with the B27 structure has higher chemical stability compared to that with the Bf structure in Ti_2_AlNb-based composites. As displayed in Figure 3b and Figure 4a, the B27-(Ti, Nb)B reinforcement is imperceptible in the matrix area. For the composite with 3.6 vol% (Ti, Nb)B, the mean length and width of the boride are 13.8 and 1.9 μm, which is lower than those of the composite fabricated by the HIP (the mean length and width of the boride of the HIP composite are 158 and 22 μm, respectively [20]). Previous research has demonstrated that the SPS possesses the features of fast heating, fast cooling and fast sintering [11,36]. These features significantly refined the dimension of (Ti, Nb)B reinforcement.

**Figure 5:**
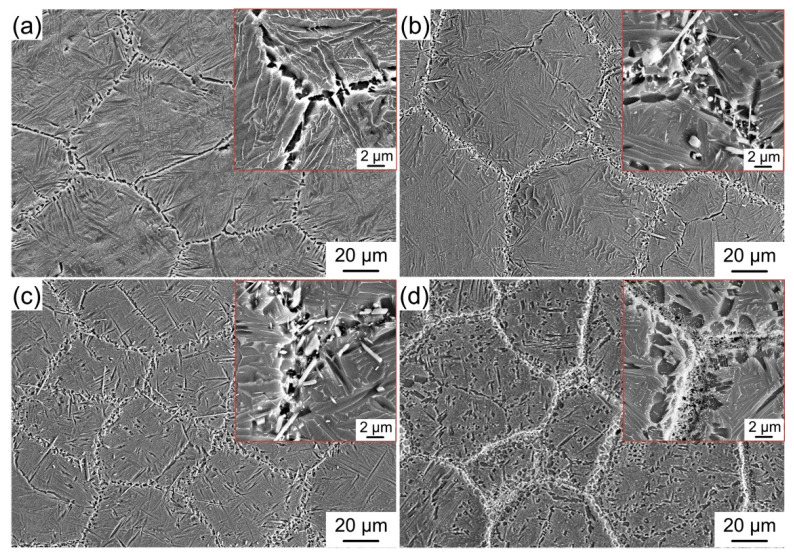
Microstructure of (Ti, Nb)B/Ti_2_AlNb composites with different (Ti, Nb)B contents (the red box exhibit the microstructure of PPBs area). (**a**) 0 vol%, (**b**) 1.6 vol%, (**c**) 3.2 vol%, (**d**) 4.9 vol%.

**Figure 6:**
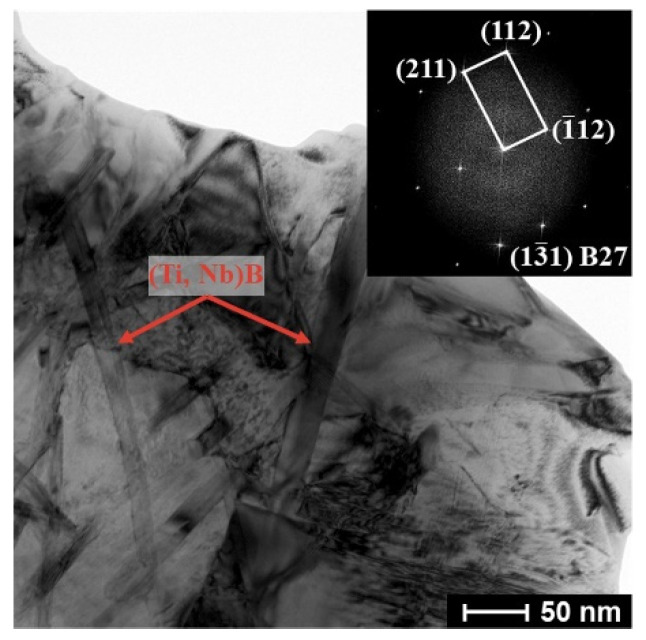
Bright-field image and corresponding FFT pattern of PPBs area in 4.9 vol% (Ti, Nb)B/Ti_2_AlNb composite.

Figure 5 exhibits the microstructure of the (Ti, Nb)B/Ti_2_AlNb composites with different (Ti, Nb)B contents. For traditional boride-reinforced Ti-based or Ti_2_AlNb-based composites with a uniform reinforcement distribution, the synthesized boride has proved to be an effective refiner of the prior β/B2 grain of the matrix [19,25]. However, for the composite fabricated in this study, the reinforcement segregates at the PPBs area and presents a near-network distribution, and thus, cannot effectively refine the prior B2 grains inside its enclosed matrix area. Moreover, the (Ti, Nb)B volume fraction displays an upward trend, with the LaB_6_ content in the original mixed powder increasing. When the LaB_6_ additive amount reaches 2.4 wt%, the (Ti, Nb)B reinforcements lose their needle-like morphology and agglomerate in the PPBs area, and the agglomerate (Ti, Nb)B reinforcement is entirely surrounded by the continuous precipitation of the brittle α_2_ phase. According to the bright-field image and FFT pattern of the PPBs area, depicted in Figure 6, the (Ti, Nb)B reinforcement in the 4.9 vol% (Ti, Nb)B/Ti_2_AlNb composite also possesses the B27 crystal structure; nonetheless, the (Ti, Nb)B grows inadequately during the sintering process, leading to the reduction in both width and length when compared with the boride synthesized in the 3.2 vol% (Ti, Nb)B/Ti_2_AlNb composite (see Figure 4b,c). The local volume fraction of the (Ti, Nb)B reinforcement *V*_L_ in the PPBs area can be estimated by:

(2) $$V_{L}=\frac{\frac{4}{3}\pi {\left(\frac{D_{m}}{2}\right)}^{3}\cdot V_{(\mathrm{Ti},\;\mathrm{Nb})B}}{\frac{4}{3}\pi {\left(\frac{D_{m}}{2}\right)}^{3}-\frac{4}{3}\pi {\left(\frac{D_{m}-D_{r}}{2}\right)}^{3}}=\frac{D_{m}{}^{3}\cdot V_{(\mathrm{Ti},\;\mathrm{Nb})B}}{D_{m}{}^{3}-{{(D}_{m}-D_{r})}^{3}}$$

where *D*_m_ is the average size of the raw Ti_2_AlNb powder, *V*_c_ and *D*_r_ are the (Ti, Nb)B volume fraction and the length of (Ti. Nb)B reinforcement, respectively. The length of the (Ti, Nb)B is measured by SEM and the bright-field image of the PPBs area, and the average size of the Ti_2_AlNb powder is 92 μm. Thus, the local (Ti, Nb)B volume fraction can be evaluated, as shown in Table 2. Due to the insufficient length, the spatial span of (Ti, Nb)B in the 4.9 vol% composite is the smallest compared with other composites, resulting in the excessive local (Ti, Nb)B content (76.8 vol%).

In addition, according to Figure 5, it also can be found that the α_2_-precipitated amount in the matrix area increases with the increase of the (Ti, Nb)B content. The α_2_ precipitation can only be perceived in the PPBs area for the composites with 1.6 and 3.2 vol% (Ti, Nb)B, while, in the 4.9 vol% (Ti, Nb)B/Ti_2_AlNb composite, the evident α_2_ precipitation can be observed in both the matrix and PPBs areas. Al has been proven to be one of the α_2_ stabilizing elements for Ti_2_AlNb-based alloys [6,37]. The chemical reaction between LaB_6_ and Ti_2_AlNb consumes the Ti and Nb element, causing the enrichment of the Al element in and around the PPBs area so that the α_2_ precipitation is promoted, especially in the PPBs area. Moreover, previous research has demonstrated that the (Ti, Nb)B maintains a coherent interface with the α_2_ precipitation and can act as an effective substrate in promoting the heterogeneous nucleation and preferred precipitation of the α_2_ phase [30]. The two reasons mentioned above account for the fact that the brittle α_2_ phase has a trend to precipitate preferentially in the PPBs area.

### 3.2. Mechanical Properties of (Ti, Nb)B/Ti_2_AlNb Composites

**Figure 7:**
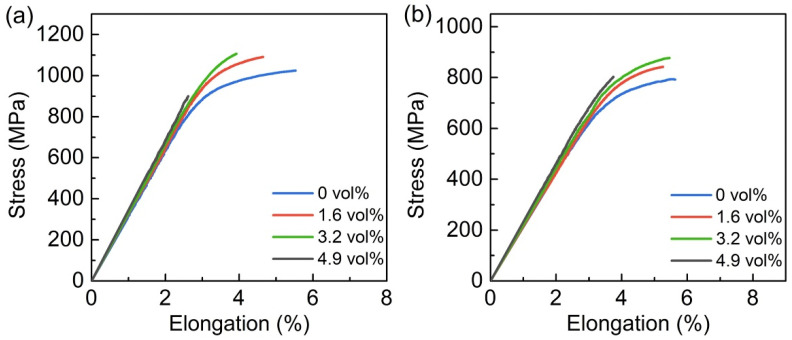
Tensile curves of (Ti, Nb)B/Ti_2_AlNb composites with different (Ti, Nb)B contents at different temperatures. (**a**) 25 °C, (**b**) 650 °C.

Figure 7 demonstrates the 25 and 650 °C tensile curves of the (Ti, Nb)B/Ti_2_AlNb composite with various (Ti, Nb)B contents. The yield strength shows an upward trend with the (Ti, Nb)B volume fraction increasing, while the elongation exhibits an opposite trend. The 25 °C tensile curve of the composite with 4.9 vol% (Ti, Nb)B possesses no obvious plastic deformation stage, displaying brittle fracture characteristics, as shown in Figure 7a. The superabundant (Ti, Nb)B deteriorates the tensile strength of the (Ti, Nb)B/Ti_2_AlNb composite caused by the premature fracture. When the environment temperature reaches 650°C, the yield strength of the composite is improved with the increasing of the (Ti, Nb)B content (see Figure 7b), which implies that the (Ti, Nb)B still exhibits an excellent strengthening effect at such an elevated temperature. In addition, with the test temperature rising, the tensile ductility of the (Ti, Nb)B/Ti_2_AlNb composite with a 3.2 and 4.9 vol% (Ti, Nb)B addition has a significant improvement. Overall, the composite with the 3.2 vol% reinforcement has optimal mechanical properties. The yield strengths of the composite at 25 and 650 °C are 998.3 and 774.9 MPa, showing an 11.8% and 9.2% improvement when compared with the Ti_2_AlNb-based alloy.

**Figure 8:**
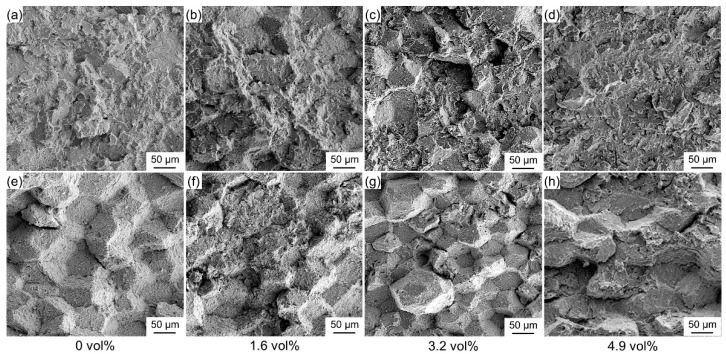
Fracture morphology of composites with various (Ti, Nb)B contents at different temperatures. (**a**–**d**) 25 °C, (**e**–**h**) 650°C.

Figure 8 sketches the fracture surfaces of the (Ti, Nb)B/Ti_2_AlNb composite at 25 °C. As shown in Figure 8a, the fracture surface of the Ti_2_AlNb alloy exhibits a transgranular fracture feature, i.e., the crack mainly propagates along the interior of the particle. For the composites with a 1.6 and 3.2 vol% (Ti, Nb)B content, the crack propagates along both the PPBs area and the interior of the previous particles, and hence, the fracture surface of the two composites exhibits the mixed feature of transgranular and intergranular fracture. When (Ti, Nb)B reinforcement agglomerates are in the PPBs area, the fracture surface also presents an entirely transgranular fracture feature; however, the fracture surface is relatively flat compared to that of the Ti_2_AlNb alloy, as displayed in Figure 8d.

Figure 8e reveals that the intergranular fracture becomes dominant for the Ti_2_AlNb alloy when the environment temperature reaches 650 °C, which suggests that the grain boundary bonding strength of the alloy weakens and becomes the key factor determining the tensile strength. Compared with the Ti_2_AlNb alloy, the fracture surface of the (Ti, Nb)B/Ti_2_AlNb composites still presents the mixed fracture feature, though only a few areas present the intergranular fracture characteristics, as shown in Figure 8f–h. The (Ti, Nb)B penetrates the PPBs area and connects two adjacent B2 grains like a needle, which can effectively inhibit the weakening of the B2 grain boundary at high temperatures.

### 3.3. Relationship between Microstructure and Mechanical Properties

Based on the results and discussion above, we can infer that the LaB_6_ mainly affects the properties of (Ti, Nb)B/Ti_2_AlNb composites by altering the α_2_-precipitated amount and the morphology as well as the synthesized amount of (Ti, Nb)B in the PPBs area. For convenience, the effect of these two factors on the mechanical properties is discussed separately.

#### 3.3.1. α2-Precipitated Amount

As shown in Figure 4c, the brittle α_2_ is prone to precipitate around (Ti, Nb)B. With the LaB_6_ addition increasing, the α_2_-precipitated amount in the PPBs area increases and the α_2_/(Ti, Nb)B interface becomes the domain matrix/(Ti, Nb)B interface. The interfacial bonding strength between the reinforcement and matrix is one of the key factors determining both the mechanical properties and fracture behaviors of composites, and generally, the prematurely interfacial debonding will emerge when the bonding strength is insufficient, leading to the low ductility of composites [38,39]. Previous results have revealed that the work of adhesion of α_2_/(Ti, Nb)B, O/(Ti, Nb)B and B2/(Ti, Nb)B are 6.41, 6.08 and 5.86 J/m^2^, respectively [30]. Thus, the difference in the bonding strength of the three interfaces is small, and the change in the reinforcement/matrix interfacial type caused by various LaB_6_ additions is not the reason for the difference in the mechanical properties of the composites.

The α_2_ phase possesses a close-packed hexagonal crystal structure (P6_3_/mmc), while the structures of the O and B2 phases are cubic ($Pm\overset{-}{3}m$) and orthorhombic (Cmcm), respectively. Among the three phases, the α_2_ phase is generally considered as being a brittle phase since it has the least independent slip system and the worst plasticity [40,41]. When the environment reaches 650 °C, the independent slip system of brittle α_2_ is still less than five, which obviously does not meet the Von Mises plastic deformation criterion [42,43]. The O phase possesses improved ductility when compared to the α_2_ phase since the <2c+a> slip system can be activated due to the low critical shear stress for slip activation [37,44]. The elasticity moduli of O, B2, α_2_ and (Ti, Nb)B are 148.0 [45], 132.2 [30], 151.7 [46,47] and 453 GPa [30], respectively. The stiffness of the PPBs area, which is full of α_2_ and (Ti, Nb)B phases, is higher than the matrix area, resulting in the stress concentration of the PPBs area during the tensile process. The insufficient deformability of the α_2_ and (Ti, Nb)B-phase means the stress concentrated in the PPBs area cannot be dissipated by deformation, causing the composites to exhibit the feature of transgranular fracture in the fracture surface, as shown in Figure 8b,f. The higher LaB_6_ addition, the higher the local volume fraction of brittle α_2_ and (Ti, Nb)B in the PPBs area, the worse the deformability of the PPBs area, and the more obvious transgranular fracture characteristics in the fracture surface, as demonstrated in Figure 8.

**Figure 9:**
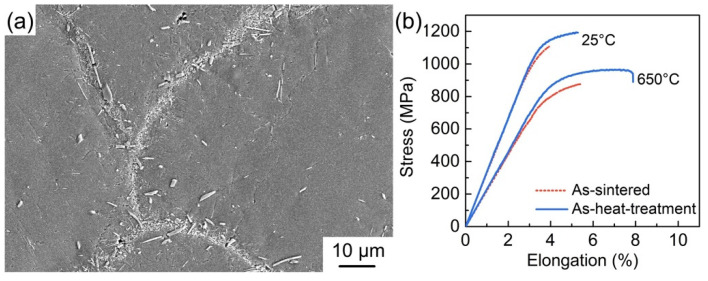
(**a**) Microstructure and (**b**) tensile curves of 3.2 vol% (Ti, Nb)B/Ti_2_AlNb composite after heat treatment.

Based on the above analysis, it can be inferred that the excessive precipitation of the brittle α_2_ phase arising from the promoting effects of the (Ti, Nb)B formation on the α_2_ precipitation is one of the main reasons for the premature failure of the PPBs area. To prove this speculation, heat treatments are used to inhibit superabundant precipitation of the α_2_ phase in the PPBs area. According to the basic theory of heat treatments, shortening the soaking time in the phase zone (where the α_2_ phase is located) is an effective method to avoid the α_2_ precipitation. Thus, the heat treatment process is set as follows: soaking at the B2 single-phase region (1100 °C) for 30 min followed by aging at the B2+O phase region (800 °C) for 600 min. After the heat treatment, the precipitation of the α_2_ phase is totally inhibited, especially in the PPBs area, as shown in Figure 9a. Figure 9b reveals the tensile curves of the as-heat-treatment composite with 3.2 vol% (Ti, Nb)B contents; by comparison, it can be found that both the ductility and tensile strength are improved with the refrain of the precipitation of brittle α_2_ in the PPBs area.

The results and analyses mentioned above prove the fact well that the LaB_6_ addition can affect the mechanical properties and fracture behavior of composites by changing the precipitate amount of the brittle α_2_ phase in the PPBs area. The excessive precipitation of the α_2_ phase leads to the premature fracture of the PPBs area and significantly deteriorates the mechanical properties of the (Ti, Nb)B/Ti_2_AlNb composites both at room and high temperatures.

#### 3.3.2. (Ti, Nb)B Morphology and Synthesized Amount

The main mechanisms by which short fiber reinforcement improves the strength of discontinuously reinforced composites, according to the strengthening theory, are dislocation strengthening and load-bearing strengthening [48,49]. As shown in Figure 5, the volume fraction of the (Ti, Nb)B reinforcement in the composite increases gradually as the LaB_6_ content increases. As shown in Figure 7, the strength of the (Ti, Nb)B/Ti_2_AlNb composite increases with increasing (Ti, Nb)B volume fraction when the LaB_6_ content is less than 1.6 wt%. This result is consistent with the results of the aforementioned study on Ti-based composites, where the higher the volume fraction of the reinforcement, the greater the strengthening effect [50,51]. Cowen et al. [20] fabricated the TiB/Ti_2_AlNb composite with a uniform reinforcement distribution and found that the 5.6 vol% TiB addition obviously degraded the mechanical properties of the composite whose fracture surface displayed a completely brittle feature at both 25 and 650 °C. Based on Cowen’s results, it can be inferred that the 3.2 vol% (Ti, Nb)B/Ti_2_AlNb composite ought to exhibit a wholly brittle feature on the fracture surface, given that the local volume fraction of the PPBs area is higher than 5.6 vol% (see Table 2). The tensile curves of the composite (as shown in Figure 7), however, exhibit a significant plastic deformation stage, indicating that tailoring reinforcements into a three-dimensional network distribution can improve the ductility of the composite when compared with the uniform distribution of reinforcement.

**Figure 10:**
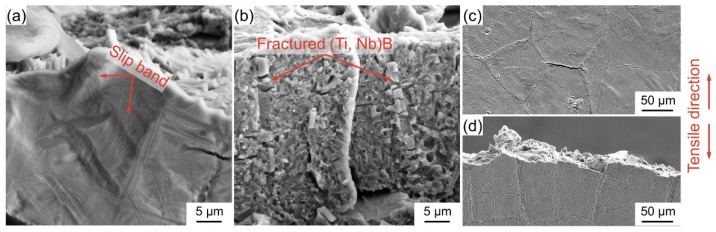
Morphology of the area adjacent to the fracture surface cracked at 25 °C. (**a**,**b**) 3.2 vol% composite, (**c**,**d**) 4.9 vol% composite.

In fact, the (Ti, Nb)B/Ti_2_AlNb composites with this novel architecture are composed of the soft area (the matrix area) and the stiff area (reinforcement enriched area), as displayed in Figure 3. The appearance of the slip band indicates the activation of the slip system, and generally, the more pronounced the slip band is, the higher the plastic deformation capacity of the material [52,53]. As illustrated in Figure 10a, abundant slip bands emerge on the matrix area adjacent to the fracture surface, which suggests that the soft area provides a better deformation ability. In addition, the cracking of boride by effective load bearing can be perceived (see Figure 10b), indicating that the stiff area offers a superior strengthening effect [54]. As a result of the synergy of the stiff and soft areas, a composite with superior overall qualities can be obtained [55,56]. When the LaB_6_ addition reaches 2.4 wt%, the boride grows insufficiently and accumulates in the PPBs area (as illustrated in Figure 3), causing an inability of the deformation to be transmitted from one particle to another. As a result, stress is severely concentrated in the PPBs area, and the crack initiates in the junction area of two particles (i.e., the PPBs area), as shown in Figure 10c. Once a crack initiates, it can spread rapidly along the composite inside (see Figure 10d), leading to premature fracture (as presented in Figure 8d).

Altering the (Ti, Nb)B volume fraction and morphology is another approach for the LaB_6_ addition to affecting the mechanical properties and fracture behaviors of (Ti, Nb)B/Ti_2_AlNb composites. When (Ti, Nb)B growth is sufficient, the strength increases with increasing LaB_6_ content; while, when the LaB_6_ addition is sufficient to induce the (Ti, Nb)B agglomeration in the PPBs area, the mechanical properties of the composites deteriorate dramatically.

## 4. Conclusions

This study focuses on revealing the effect of the (Ti, Nb)B content on the microstructure and mechanical properties of in situ (Ti, Nb)B/Ti_2_AlNb composites with tailored three-dimensional network architecture. Several conclusions can be drawn as follows:

(1) The (Ti, Nb)B/Ti_2_AlNb composites with tailored three-dimensional network architecture, in which the boride crystalized into a B27 crystal structure and gathered in the prior article boundaries (PPBs) area, was successfully fabricated by ball milling and subsequent spark plasma sintering.
(2) The synthesized content of boride altered the α_2_-precipitated amount and the (Ti, Nb)B morphology. When the boride content reached a specific value, the (Ti, Nb)B reinforcement agglomerated in the PPBs area due to insufficient growth, and the brittle α_2_ phase precipitated continuously adjacent to the reinforcement.
(3) When the (Ti, Nb)B content reached a reasonable range, the yield strength of the composites improved and the ductility exhibited an opposite trend with the increase in the volume fraction of reinforcement. The (Ti, Nb)B incorporation inhibited the strength weakening of the PPBs area at high temperatures, which significantly increased the strength of the composites.
(4) By reducing the α_2_-precipitated content in the PPBs area and avoiding agglomeration of boride by increasing the (Ti, Nb)B growth length, these were both effective methods to improve the mechanical properties of the composite with a high volume fraction of reinforcement.

## Figures and Tables

**Table 2:** Local (Ti, Nb)B volume fraction in PPBs area for composites with different (Ti, Nb)B contents.

*V*_(Ti,Nb)B_ (vol%)	*D*_m_ (μm)	*D*_r_ (μm)	*V*_L_ (vol%)
0.0	92.0	—	—
1.6		15.5	3.8
3.2		13.8	8.3
4.9		2.0	76.8

## Data Availability

The data presented in this study are available on request from the corresponding author.
